# Botulinum Toxin Injection for Treatment of Chronic Anal Fissure: Is There Any Dose-Dependent Efficiency? A Meta-Analysis

**DOI:** 10.1007/s00268-016-3693-9

**Published:** 2016-08-18

**Authors:** Adam Bobkiewicz, Wojciech Francuzik, Lukasz Krokowicz, Adam Studniarek, Witold Ledwosiński, Jacek Paszkowski, Michal Drews, Tomasz Banasiewicz

**Affiliations:** 1 Department of General, Endocrinological Surgery and Gastroenterological Oncology, Poznan University of Medical Sciences, Przybyszewskiego 49, 60-355 Poznan, Poland; 2 Department of Dermatology, Venerology and Allergology, Charité - Universitätsmedizin Berlin, Charitéplatz 1, 10117 Berlin, Germany

## Abstract

**Background:**

Chronic anal fissure (CAF) is a linear split of the anoderm. The minimally invasive management of CAF such as botulinum toxin (BT) injection is recommended. However, the exact efficient dose of BT, number of injections per session and the injection sites are still debatable. The aim of this analysis was to assess the dose-dependent efficiency of botulinum toxin injection for CAF.

**Methods:**

PubMed and Web of Science databases were searched for terms: “anal fissure” AND “botulinum toxin.” Studies published between October 1993 and May 2015 were included and had to meet the following criteria: (1) chronic anal fissure, (2) prospective character of the study, (3) used simple BT injection without any other interventions and (4) no previous treatment with BT.

**Results:**

A total of 1577 patients from 34 prospective studies used either Botox or Dysport formulations were qualified for this meta-analysis. A total number of BT units per session ranged from 5 to 150 IU, whereas the efficiency across analyzed studies ranged from 33 to 96 %. Surprisingly, we did not observe a dose-dependent efficiency (Spearman’s rank correlation coefficient, *ρ* = 0.060; *p* = 0.0708). Moreover, there were no BT dose-dependent postoperative complications or fecal incontinence and significant difference in healing rates compared BT injection into the anal sphincter muscles.

**Conclusions:**

BT injection has been an accepted method for the management of CAF. Surprisingly, there is no dose-dependent efficiency, and the postoperative incontinence rate is not related to the BT dosage regardless the type of formulation of botulinum neurotoxin used. Moreover, no difference in healing rate has been observed in regard to the site and number of injections per session.

## Introduction

An anal fissure is defined as a tear in the skin within the distal anal canal. A chronic anal fissure (CAF) usually occurs in young adults and affects both sexes equally and is predominantly localized at the posterior commissure of the anal canal which is possibly associated with local hypoperfusion at the posterior midline [1, 2]. Although the exact pathogenesis of anal fissure is still unclear, based on recent studies, a local ischemia seems to play a crucial role in the pathogenesis. The increased resting anal tone of internal sphincter muscle influences prolonged compression of anodermal arteries passing through the muscle, resulting in local ischemia [3, 4].

Lateral internal sphincterotomy primary described by Eisenhammer has been widely accepted as a gold standard surgical procedure for chronic anal fissure. However, the potential risk of postoperative complications including gas or stool incontinence is caused by the fact that recently there has been more focus on less invasive management such as chemical sphincterotomy of CAF [5, 6]. The use of varying topical pharmacological agents such as isosorbide dinitrate, glyceryl trinitrate, calcium antagonists or muscarinic agonists has been used with varying healing rates [7–9]. However, the fundamental drawbacks of these therapies are short-term effectiveness and potential side effects [10, 11]. Recently, botulinum toxin (BT) has been adopted for the treatment of chronic anal fissure. An injection of BT leads to the blockade of acetylcholine release and causes short-term paralysis of internal sphincter muscle, resulting in a reduction in anal tone [12, 13].

Although this method of treatment has been used worldwide, a firm recommendation for the injection of BT is still under debate. Moreover, there are no broadly established intervals between further injections and how many injections should be made at one session. There is limited information about how the dose of BT influences the potential postoperative gas or stool incontinence.

The main goal of the study was to answer crucial questions: (1) Is there any dose-dependent efficiency of BT for CAF, (2) Is there any correlation between doses of BT and potential complication rate including incontinence, (3) Is there any correlation between the site of injection (internal or external anal sphincter muscle) or the number of injections and the efficiency of BT injection for CAF.

## Materials and methods

### Data sources and search strategy

The study was performed at tertiary reference teaching hospital. A comprehensive literature search was performed independently by four investigators. PubMed and Web of Science database were searched for terms: “anal fissure” AND “botulinum toxin.” Only English references published before May 2015 were included into the meta-analysis.

### Inclusion and exclusion criteria

All studies included into the meta-analysis met the following criteria: (1) diagnosis of chronic anal fissure, (2) prospective character of the study, (3) simple botulinum toxin injection without any other interventions (e.g., simultaneous ointment application, lateral internal sphincterotomy and others) and (4) no previous treatment with botulinum toxin.

Studies comparing different doses of BT injection in different groups of patients were included into the study and listed separately for analysis. Either Botox or Dysport formulation of botulinum neurotoxin was included into the meta-analysis.

Studies such as: review, meta-analysis, letters to the editor, articles not in English, studies on animal models, case reports (*n* < 10 patients), conference abstracts, acute anal fissure, studies on pediatric population were excluded from the meta-analysis.

### Data inclusion

The following data were collected from each study and included: author, year of publication, number of patients, type of BT formulation, site of BT injection (internal or external anal sphincter), dose of BT injected (total number of injected BT units), injection volume (total in ml per session), number of injections per session, efficiency (%), length of follow-up (weeks), the type of follow-up (physical examination, questionnaire, manometry or others), complications (number and character) and rate of incontinence and time of resolving (weeks).

### Statistical analysis

Efficacy was described as a treatment success rate, being the number of healed patients (without clinical symptoms) divided by the number of all treated patients (without the placebo control groups), expressed in percent. All calculations regarding dose dependencies were performed using Spearman’s rank correlation test. *p* values <0.05 were considered significant. Statistical analysis was performed with the use of R statistical package.

In the purpose of appropriate outcomes interpretation, conversion factor of 1:3 for Botox equivalent unit to Dysport formulation was used.

## Results

**Fig. 1:**
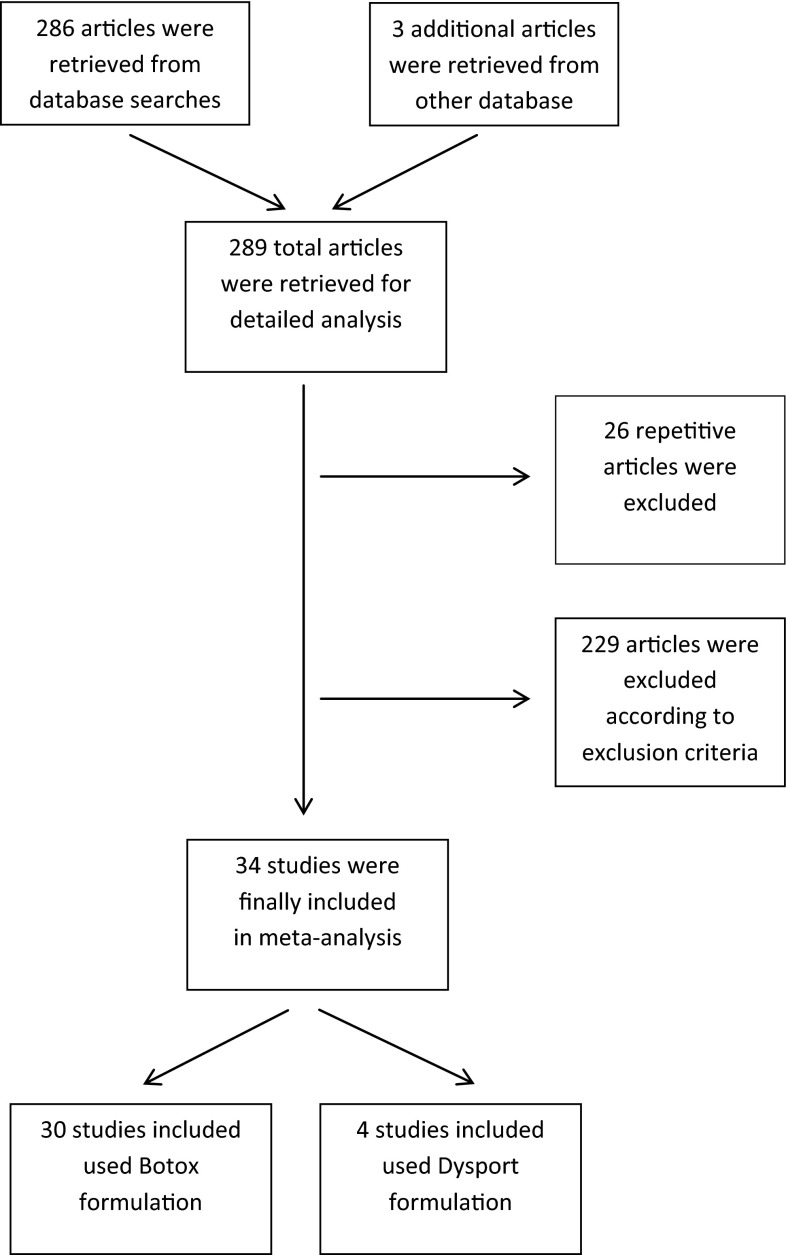
Flowchart of the study

A total of 286 studies were retrieved based on the publication search. A total of 26 citations were excluded because of duplication. A total of 198 publications were excluded from analysis because they did not meet the inclusion criteria. Finally, 34 studies were enrolled into the study meeting the criteria for meta-analysis. A total of 30 studies used Botox formulation, whereas in four studies Dysport formulation was used. The number of studies searched, evaluated and included into the meta-analysis is shown in the flowchart (Fig. 1).

A total of 1577 patients from 34 prospective studies were qualified for meta-analysis. Studies included in the meta-analysis are summarized in Table 1.

**Table 1:** Studies included in the meta-analysis

Authors and year of study	N	Site of BT injection (external-1; internal-2)	Units of BT (Units, total)	Injection volume (total, ml)	Number of injections per session	Efficiency (%)	Follow-up (weeks)	Type of follow-up (physical examination-1, per rectum examination-2 questionnaire-3, manometry-4)	Complications	Number of patient developed incontinence	Time of incontinence resolving (weeks)
Madalinski et al. 2001 [14]	13	2	50	NA	2	50	NA	1,2	NA	NA	0
Lysy et al. 2001 [15]	30	2	20	0.5	2	66	12	1,2,4	1-Perianal thrombosis	0	0
Steele et al. 2000 [16]	50	2	20	0.4	2	96	8	1,2,4	0	0	0
López et al. 1999 [17]	76	2	80	NA	2	67	12	1,2	1-Perianal thrombosis	2	NA
Maria et al. 2000 [18]	50	2	20	NA	2	74	8	1,2,4	0	0	0
Mínguez et al. 1999 [19]	23	1	10	0.4	2	83	24	1,2,4	1-Subcutaneous abscess	5	1
	27	2	15	0.6	2	78	24	1,2,4	1-Hematoma	2	1
	19	2	21	0.84	2	90	24	1,2,4	0	0	0
Brisinda et al. 1999 [20]	25	2	20	0.4	2	96	8	1,2,4	0	0	0
Jost et al. 1999 [21]^a^	25	2	10	0.2	2	76	12	1,2,4	0	1	2
	25	2	20	0.2	2	80	12	1,2,4	0	3	2
Maria et al. 1998 [22]	23	2	15	0.3	2	44	8	1,2,4	0	1	1
	34	2	20	0.4	2	67	8	1,2,4	0	0	0
Maria et al. 1998 [23]	15	2	15	0.4	2	73	8	1,2,4	0	0	0
Jost et al. 1996 [24]	12	2	5	0.2	2	75	24	NA	0	0	0
Jost et al. 1999 [25]	20	1	5	0.2	2	70	12	1,2,4	0	0	0
	30	1	10	0.4	2	63	12	1,2,4	0	2	2
Asim et al. 2014 [26]	21	2	20	NA	2	57	12	1,2,3	0	3	6
Berkel et al. 2014 [27]^a^	27	2	60	0.3	2	67	9	1,2,3	0	5	3
Valizadeh et al. 2001 [28]	25	2	50	1	2	44	48	1,2,3	0	0	0
Vanella et al. 2012 [29]	3	1	20	0.2	2	NA	8	1,2,4	0	0	0
	11	1	30	0.3	2	NA	8	1,2,4	0	0	0
	46	1	50	0.5	2	65	8	1,2,4	0	1	3
Samim et al. 2012 [30]	60	2	20	0.2	2	43	12	1,2,3	1-Perianal itching	0	0
Piccini et al. 2009 [31]	60	2	30	0.45	2	48	4	1,2,3	3-Hemorhoidal thrombosis	2	NA
Alghaity et al. 2008 [32]	50	2	40	0.4	2	86	144	1,2,3	4-Hematoma, perianal abscess	10	2
Elhady et al. 2009 [33]	40	2	40	0.8	2	48	12	1,2,4	3-Hematoma	0	0
Festen et al. 2009 [34]	37	2	20	NA	2	38	16	1,2,3	0	0	0
Nasr et al. 2010 [35]	40	2	20	0.5	2	62	18	1,2,3	8-Hematoma	0	0
Brisinda et al. 2007 [36]	50	2	30	NA	2	92	8	1,2,4	0	3	3
Jones et al. 2006 [37]	15	2	25	0.4	2	67	24	1,2,4	0	2	2
De Nardi et al. 2006 [38]	15	2	20	0.4	2	33	144	1,2	0	0	0
Iswariah et al. 2005 [39]	17	2	20	NA	2	41	26	1,2	0	2	NA
Thornton et al. 2005 [40]	56	2	20	NA	2	66	12	1,2,4	1-Urinary stress inc.	3	NA
Arroyo et al. 2005 [41]	40	2	25	1	3	45	48	1,2,4	1-Hematoma	2	8
Arroyo et al. 2005 [42]	100	2	25	1	3	47	156	1,2,3,4	5-Perianal thrombosis, hemorrhoidal thrombosis	6	NA
Birisinda et al. 2004 [43]^a^	50	2	50	1	2	92	8	1,2,4	0	11	3
	50	2	150	1	2	94	8	1,2,4	0	8	3
Siproudhis et al. 2003 [44]^a^	22	2	100	0,4	2	80	12	1,2	5-Subcutaneous abscess, perianal thrombosis	0	0
Colak et al. 2002 [45]	34	2	50	1	2	70,5	8	1,2,4	0	NA	0
Mentes et al. 2003 [46]	61	2	30	NA	2	74	8	1,2,3	0	0	0
Birisinda et al. 2002 [47]	75	2	20	0.4	2	89	8	1,2,4	0	NA	0
	75	2	30	0.6	2	96	8	1,2,4	0	5	2

*NA* not available

^a^Four studies used DYSPORT formulation of botulinum neurotoxin [Refs. 21, 27, 43, 44]

In majority of studies (*n* = 31, 91.2 %), preferably internal anal sphincter was injected. In all studies used Dysport formulation, only internal anal sphincter was injected, whereas in three studies with Botox formulation authors injected external anal sphincter. Usually, only two site of anal sphincter was preferred to be injected per session, whereas in two studies (Botox formulation), three sites of anal sphincter were injected with BT.

The results of BT treatment were evaluated based on physical examination (including per rectum examination) in 33 studies, questionnaire (usually self-evaluation of pain, bleeding and incontinence) in 10 studies and anorectal manometry in 19 studies. In one study, there was no explanation of methods.

The mean time of follow-up ranged from 4 to 156 weeks after BT injection. The mean time of follow-up was significantly shorter when compare Botox *versus* Dysport formulation of botulinum neurotoxin used (28.9 ± 42.6 vs 10.2 ± 2.1 weeks).

**Fig. 2:**
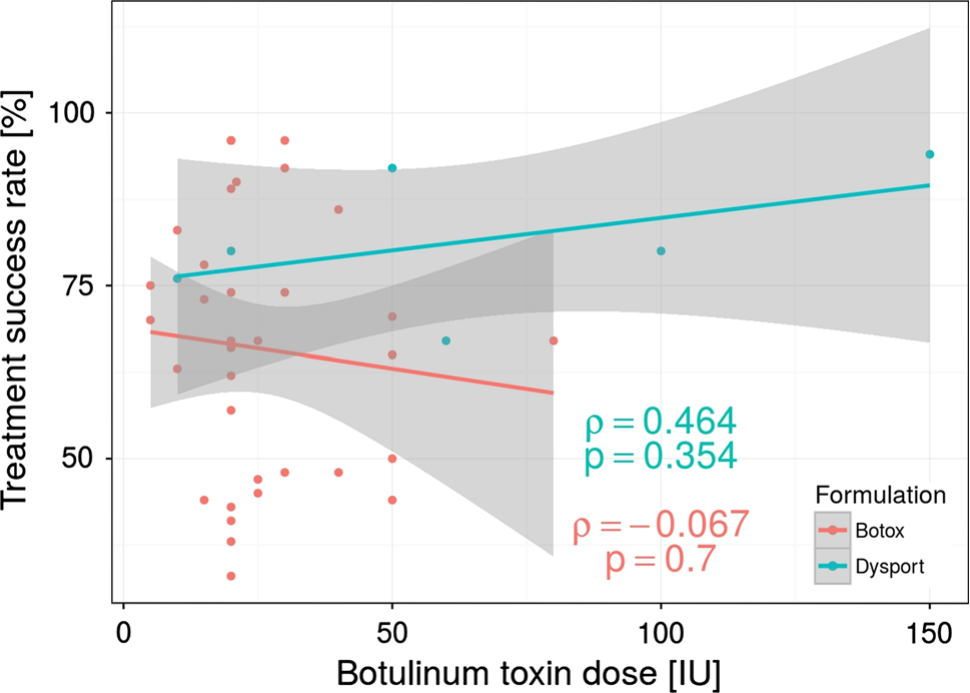
Botulinum toxin injection dose used in the clinical studies in relation to the treatment success rate (defined as the percentage of positive treatment outcomes in all patients treated with BT) using two type of BT formulations (Botox and Dysport). Note the lack of dose dependency in positive therapy results. Each *dot* represents an independently treated patient group

**Fig. 3:**
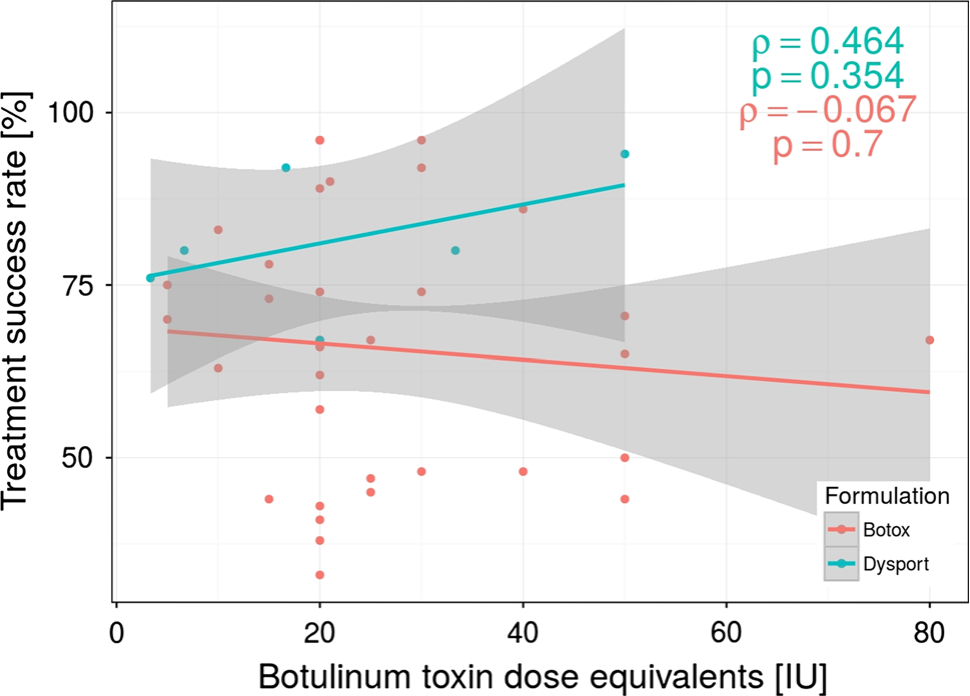
Botulinum toxin injection dose used in the clinical studies in relation to the treatment success rate after conversion factor used (Dysport/Botox equivalency ratio of 3:1)

A total of BT units per session ranged from 5 to 80 IU and 10–150 in Botox and Dysport formulation groups, respectively. The efficiency across analyzed studies varied from 33 to 96 % and from 67 to 94 %, respectively, for Botox and Dysport groups. The meta-analytical correlation between the amounts of BT units injected (analyzed separately for Botox and Dysport group) and its efficiency is illustrated in Fig. 2. Based on Spearman’s rank correlation, there was no significant statistical correlation. Moreover, there was no significant statistical correlation when we used conversion factor of 1:3 for Botox equivalent unit to Dysport formulation (Fig. 3).

**Fig. 4:**
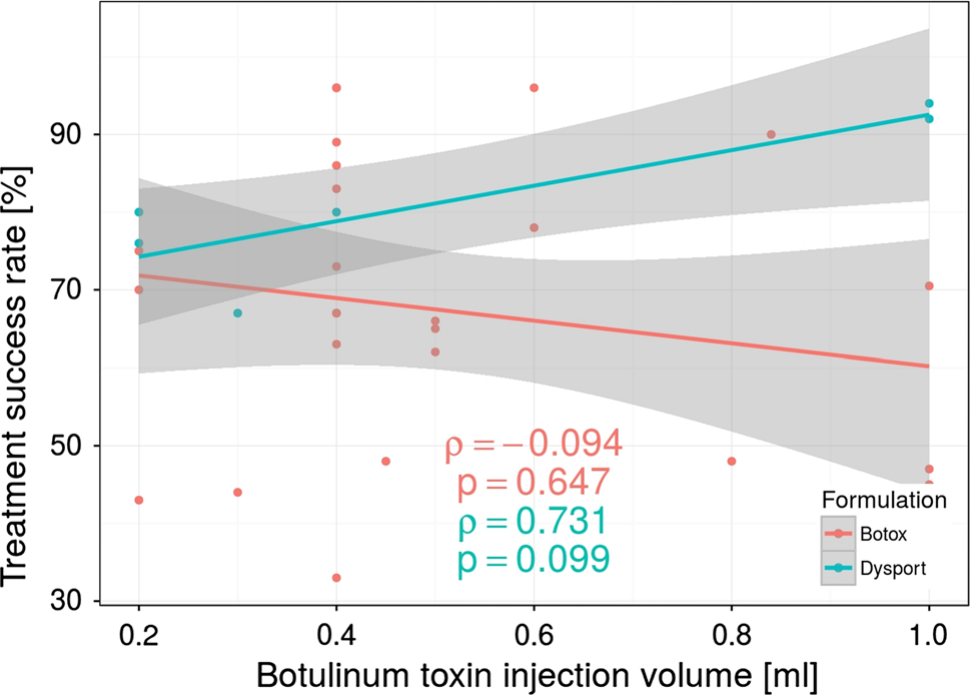
Botulinum toxin injection volume used (Botox and Dysport formulations) in the clinical studies in relation to the treatment success rate. Note the lack of correlation positive therapy results. Each *dot* represents a group of patients treated with the same volume of the botulinum toxin but does not imply separate studies

A total mean volume of injected BT (both Botox and Dysport) ranged from 0.2 to 1.0 ml per session. There was no statistical correlation between total volume of BT injected and efficiency based on Spearman’s rank correlation (Fig. 4).

**Fig. 5:**
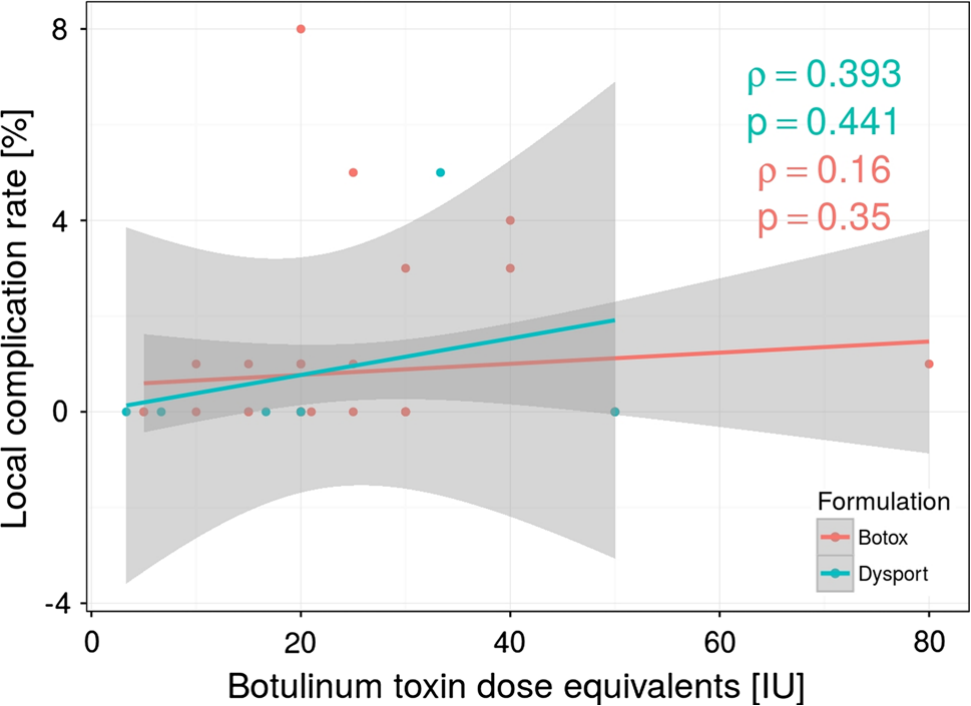
Lack of correlation between the botulinum toxin doses related to the number of local postoperative complications regarding Botox and Dysport formulations used (Dysport/Botox equivalency ratio of 3:1)

A total of 35 (2.22 %) patients developed local postoperative complications such as hematoma, perianal thrombosis or perianal abscess as the most common ones. In fourteen studies, authors reported local complications, which ranged from one to eight per study. When we analyzed the amount of BT injections and the number of complications, we did not find any statistical correlation (Fig. 5). There was also no significant difference in postoperative complication rate between Botox and Dysport groups.

**Fig. 6:**
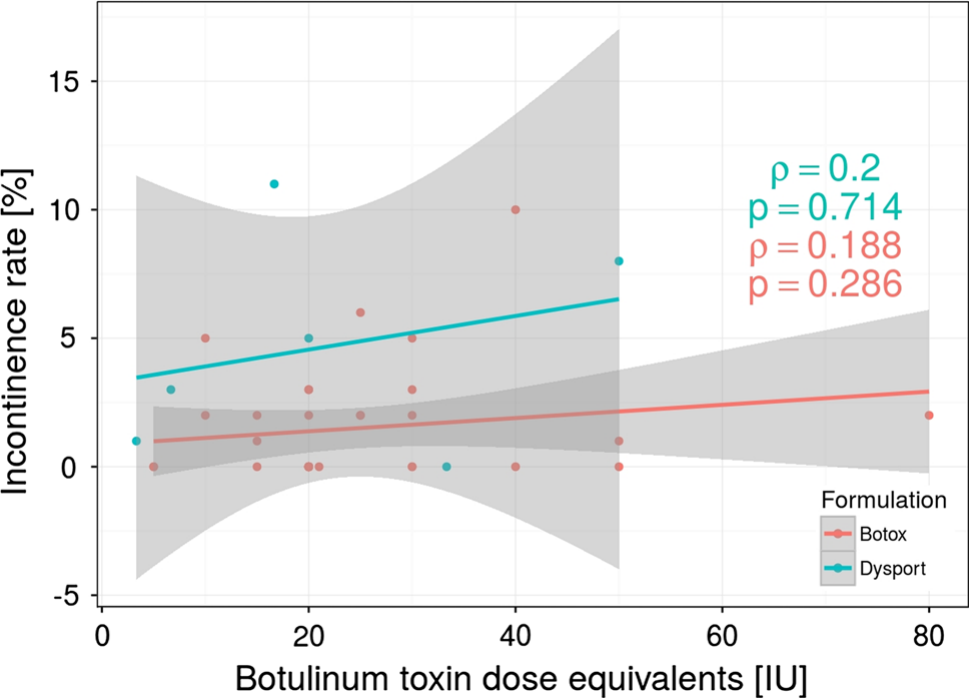
Botox and Dysport (Dysport/Botox equivalency ratio of 3:1) dose injection does not correlate with the rate of postoperative fecal incontinence

Seventy-nine (5.01 %) patients reported transient fecal incontinence, 51 patients in Botox group and 28 in Dysport group. However, in all cases symptoms resolved within a couple of weeks (ranged from 1 to 8 weeks). Surprisingly, we did not find any statistical correlation between amount of BT injection (units) and frequency of incontinence (Fig. 6). Moreover, there was no significant correlation between BT units and the time required for the resolution of incontinence caused by BT injection. There was also no significant difference when compare Botox and Dysport groups regarding incontinence rate.

In three studies with a total of 133 patients, BT was injected within the external anal sphincter, whereas in remaining thirty-one studies internal anal sphincter was preferably injected with BT. There was no significant difference in efficiency concerning the site of injection.

## Discussion

A botulinum toxin (BT) injection has been widely accepted as the method of CAF treatment. High efficiency of BT injection for CAF was confirmed in many studies [43, 48–50]. However, based on these studies there are still some controversies regarding the optimal dosage of BT and its influence on efficiency of the CAF treatment. This meta-analysis showed that there is no dose-dependent efficiency of BT injection. Although the total number of injected units of BT ranged between 5 and 150 units per session, there was no statistical difference in the treatment success rate concerning the dosage of BT, for either Botox or Dysport formulations. However, some authors observed dose-dependent healing rates [19, 47]. Vinella et al. [29] reported no significant effect of different BT doses (20, 30 and 50 IU) and the site of injection on healing rate. Moreover, these results are in line with randomized controlled trial presented by Brisinda et al. [43] compared Botox versus Dysport formulations. Using a conversion factor of 1:3 for different BT formulations, there was no significance in healing rate. Based on current studies, the explanation of comparable results of CAF efficiency regardless the applied dosage of BT is unsolved. Some authors indicated diffusion of BT as an important factor which may boost the mechanism of action within target tissue [51–53]. Based on histologic staining of acetylcholinesterase, equal distribution of biological effect of BT was observed within the entire studied muscle when high dose of BT was applied, whereas in other experimental scenarios with low dose of BT injection, a gradient down the muscle was observed [51–53]. Diffusion of BT was also suggested as the most likely reason for no observable difference in healing rate using different BT dosages in CAF treatment [54]. Although some other factors such as presence of antibodies, cholinergic cells susceptibility to botulinum toxin or binding and/or internalizing properties of cholinergic cells have been proposed as a rationale explanation for varying clinical response to BT treatment, there is still no firm evidence support these hypothesis [43].

Only 2.2 % of patients (*n* = 35) developed perianal complications due to BT injection based on presented meta-analysis. Thus, this result confirmed the advantage of BT injection regarding postoperative complications when compared to lateral internal sphincterotomy (LIS) [55]. Chen et al. [55] concluded that LIS is superior to BT for CAF considering healing rate and recurrence rate. However, BT injection was recommended for patients who either refuse surgical management, were previously treated with LIS or are at risk of developing postoperative incontinence (such as women with many vaginal deliveries, previous anal surgery or previously diagnosed incontinence). The overall incontinence rate based on this meta-analysis was established of 5.01 % (*n* = 79). Importantly, majority of patients reported only transient flatus or mucus incontinence that lasted up to 8 weeks postoperatively and disappeared spontaneously.

In majority of studies, only internal sphincter was injected. Explanation of such management is based on the underlying pathology of CAF associated with internal sphincter involvement. However, in some studies external sphincter muscle was preferably injected, supported by the study indicating external anal sphincter muscle as a major contributor to the pathogenesis of CAF which seems to be precisely the opposite to other studies [21, 56]. Regardless of whether internal or external sphincters are injected, the results of healing rate are comparable. Possibly, BT diffuses into the internal anal following external sphincter injection and vice versa which was stated by Jost et al. [21, 56].

Recently, the attention has been paid to the psychological aspect in patients with CAF, resulting in varying responses to treatment. The high correlation with mental components was evidenced by significantly higher incidence of CAF in patients with type D (distressed) personality (defined as the co-occurrence of negative affect and social inhibition) [57]. While this type of personality generally represents 16 % of the population, it raises up to 33 % of in patients with CAF. In this group of patients, the symptoms associated with CAF are more easily recognized what may impair the quality of their life more than in patients with other type of personality [57]. It is also important to note that the placebo effect in patients treated for CAF is relatively high. Berry et al. [58] conducted placebo-controlled study comparing nitroglycerin (NTG) 0.4 % ointment with placebo in patients with CAF. Surprisingly, the reduction in pain (defined as at least 50 % due to visual analog scale, VAS) was achieved by 72.4 % in the NTG group compared to 64.5 % in the placebo group. Moreover, side effects (including headaches) were reported by 69.9 % of patients treated with nitroglycerin ointment but also by 47.6 % patients in the placebo group. The use of BT injection for CAF as a generally recommended and efficient method of treatment is per se the important aspect of management in this group of patients, which may also explain the no dose-dependent efficiency of BT.

Although the underlying pathology of achalasia, the course of the disease and the response for BT treatment is quite different, there is also no observed dose-dependent efficiency. Based on a recent large multicenter study, the administration of 100 IU of BT (repeated 1 month later) was more efficacious than either 50 or 200 IU [59]. The same conclusion was achieved by Cuillière et al. [60]. An increased dose of BT from 100 to 200 IU did not significantly increase the success rate.

To limit the publication bias, the appropriate selection of studies was performed. However, this meta-analysis has some limitations. First, the time of outcome evaluation ranged from 4 to 156 weeks after BT injection which may possibly influence the efficiency rate. The use of BT might hasten the treatment of anal fissures. However, we did not confirm any significant correlation between the time period of BT injection and its efficiency. Second, although detailed selection of studies was performed, heterogeneity among analyzed studies exists due to a surgeon’s preference and experience as well as the heterogenic sample of patients included into the study. Third, methodology of outcome evaluation differs among studies which may influence the interpretation of the results. Although in all studies clinical examination was performed, questionnaire and anorectal methodology were performed in ten and nineteen studies, respectively. Fourth, most studies were designed not as double-blinded studies which may also impact the outcomes.

## Conclusions

Based on the present meta-analysis there is no BT dose-dependent efficiency for CAF. Moreover, postoperative incontinence rate as well as complication rate is not related to the BT dosage. There was also no difference in healing rate observed in regard to the injection site and the number of injections per session. Further studies are needed in order to evaluate the real value and optimal dose of BT for CAF management. Moreover, the firm and unequivocal criteria for the definition of CAF, end points and the evaluation of outcomes are needed in order to establish the optimal dose of BT injection for CAF treatment.
